# Prevalence and familial predictors of suicidal behaviour among adolescents in Lithuania: a cross-sectional survey 2014

**DOI:** 10.1186/s12889-016-3211-x

**Published:** 2016-07-12

**Authors:** Apolinaras Zaborskis, Dainora Sirvyte, Nida Zemaitiene

**Affiliations:** Lithuanian University of Health Sciences, Academy of Medicine, Faculty of Public Health, Institute for Health Research, A.Mickeviciaus str., 9, LT-44307 Kaunas, Lithuania

**Keywords:** Suicide, Suicidal behaviour, Adolescents, Family, Parent–child relationships, Parenting

## Abstract

**Background:**

In the past decades Lithuania has been experiencing a very high suicide rate among young people and there are scarce data on the role of the family in shaping these people suicidal behaviour. This study investigated the prevalence of suicidal ideation and attempts, as well as their association with a range of familial factors in a representative sample of Lithuanian adolescents.

**Methods:**

Study subjects (*N* = 3572) were adolescents aged 13- and 15-years from the schools in Lithuania who were surveyed in Spring 2014 according to the methodology of the cross-national Health Behaviour in School-aged Children (HBSC). A standard HBSC international questionnaire was translated into Lithuanian and used anonymously to obtain information about suicidal behaviour (stopped doing activities, considered suicide, planned suicide, and suicide attempts) and family life (family structure, quality of communication in family, parental monitoring and bonding, parenting style, family time, etc.). Logistic regression was used to assess association between suicidal behaviours and familial variables.

**Results:**

Forty three percents of surveyed adolescents reported presence of emotions that stopped doing activities during the last 12 months, 23.8 % seriously considered attempting suicide, 13.7 % made a suicide plan, 13.2 % attempted suicide, and 4.1 % needed treatment because of suicide attempt in the previous year. Adolescents from non-intact families reported more suicidal ideation (*OR* ranged from 1.32 to 1.35, *P* < 0.05) and more suicide attempts (*OR* = 1.70, 95 % *CI* 1.38-2.09, *P* < 0.001). Among adolescents from intact families, some manisfestations of suicidal behaviour were significantly associated with low satisfaction in family relationships, low father’s and mother’s emotional support, low mother’s monitoring, low school-related parental support, authoritarian-repressive father’s parenting style and permissive-neglectful mother’s parenting style, but rare family time together and rare electronic media communication with parents were inversely associated with suicidal behaviour. The boys, 15-year-olds and adolescents who indicated often activities together with their families were more likely than their counterparts to report suicide attempts treated by a doctor or nurse.

**Conclusion:**

The young people of Lithuania are at particular risk for suicides. A non-intact family structure and weak family functioning are significant predictors of suicidal ideation and attempts among adolescents of Lithuania. It is essential to consider family life practices in planning intervention programs for prevention of suicides among adolescents.

## Background

Statistics show that suicide is currently one of the leading causes of death among young and middle-aged people and represents a significant public health problem worldwide [1–3]. In many countries, it’s extremely disturbing that this issue is becoming more and more associated with the younger age groups [4–6]. Lithuania has been among the countries with the highest suicide rate for more than 20 recent years [7]. According to the statistical data of the country [8], from early 1990s, the frequency of suicide increased amongst adults and young people aged 15–19 years. After 2002, a decrease in deaths by suicide was observed both for the whole population and for young people aged 15–19 years. In 2012, age-standardized suicide mortality for the whole population was 28.3 and for the population aged 15–19 was 14.9 per 100 000 of population, therefore, suicide covered 26 and 35 % of external causes of death for all population and young people respectively [9].

Adolescence is the time of greatest risk for the first onset of suicidal behaviors, however, suicide prediction in young ages is complex and difficult [10, 11], because suicidality is considered to be a multifactorial phenomenon [12, 13]. Non-fatal suicidal behaviours, such as seriously considered attempting suicide, made a suicide plan and attempted suicide, occurs at least 10 to 20 times more than completed suicides [14]. Previous studies have reported a variety of risk factors related with suicidality in adolescence [15–17]. The relationship between psychiatric disorders (like depression) and adolescent suicide is now well established [18, 19]. Mood disorders, substance abuse and prior suicide attempts are strongly related with youth suicides [15, 19–21]. Factors related to social alienation and precipitating problems also contribute to the risk of suicide [22–24]. In order to full understanding risk factors for suicide and to develop strategies for intervention, it is important also to analyze the association between adolescents’ suicidality and their family social and psychological climate. Several studies have tried to explore the effect of parental factors [21, 25] and other factors related to family adversity [26–28]. However, the researchers of the vast majority of studies did not attend to whether the familial risk factors preceded the development of suicidal symptoms. Given the heterogeneity of study samples and designs, little conclusive evidence has been found, thus, further research is needed to replicate and determine the magnitude of effect of most familial factors.

The political and societal transition in Lithuania, like in other countries of Central and Eastern Europe, started at the beginning of the 1990s brought the painful transformation in family life: tragically declining birth rates, an increased number of divorces, changes in household composition or family structure, etc. [29, 30]. For illustration of these changes, the official census data of Lithuania indicates a drastic increase in the number of extramarital births: from 7.0 in 1990 to 22.6 in 2000, and to 25.7 in 2010 per 100 births [31]. The proportions of children growing up in a nuclear family composed of a biological father and mother – intact family – has reduced over the past decade. These transformations may have affected child rearing and socialization of children. Such family is less able to control self destructive behaviour of the children, such as smoking, alcohol and drugs intake [29], well-being and suicidal behaviour [32].

It is consequently crucial to understand how, and under what conditions, the family structure and functioning is related with the development of the young person and, especially, with health risk behaviours. The focus of this study lies in identifying the role that several familial factors play in adolescent suicidal behaviour. Furthermore, protective factors associated with the interpersonal relationships between family members are to be identified. Within this area, there are analyses of the specific variables that shape the interpersonal relationships (family dynamics) built within the family setting including: communication and attachment to parents, monitoring, and disciplinary parenting styles [33]. A review of the literature identifies that there is better adjustment in children and adolescents (e.g. less risk of suicide) who reported having an open communication with their parents, or who perceived them as physically and emotionally accessible, or who felt vigilant parental monitoring [34]. Because research in this field among the Lithuanian adolescent population is still scarce [35], there is a need to investigate how much the above mentioned findings are appropriate within the Lithuanian family. Knowledge on the familial predictors of suicidal behaviour in adolescents is necessary to inform future intervention development to reduce the mentioned trouble among young people.

The *Health Behaviour in School-aged Children (HBSC)*, a World Health Organization cross-national study, considers the family as one of the significant domains of adolescent life [33, 36–40]. The study, which started in Lithuania in 1994, provides a realistic opportunity to explore adolescent health behaviour, including non-fatal suicidal behaviour, in the family context. The recent survey that was carried out in Spring 2014 in Lithuania included full set of optional packages developed by the *Family Culture* working group [33].

The aim of this study was to investigate the prevalence of non-fatal suicidal behaviour (stopped doing activities, considered suicide, planned suicide, and suicide attempt) and its association with a range of familial factors in a representative sample of Lithuanian adolescents who were surveyed in the recent HBSC wave. We hypothesized that a non-intact family structure, weak child–parent relationships and contact, lack of parental control, etc. would be predictors of adolescent suicidal thoughts and suicide attempts.

## Methods

### Subjects and study procedures

The data analysed here were collected in the school-based, cross-sectional, anonymous survey conducted in 2014 (April–May) in Lithuania according to the methodology of World Health Organization collaborative cross-national HBSC study (more detailed information about the study is provided elsewhere [39–42]. Researchers followed the standardized international research protocol [43] to ensure consistency in survey instruments, data collection and processing procedures.

The population selected for sampling was 11-, 13- and 15-year-olds attending general school. Participants were selected using a clustered hierarchical sampling design, where the initial sampling unit was a class of the fifth, seventh or ninth grades (the most appropriate grades for required students’ age groups). Samples of students were drawn to be representative by age and gender. According to the research protocol, the recommended sample size for Lithuania was 1500 students per age group. In total, 356 classes from 129 schools from the whole country were drawn to ensure the requested number of surveyed students.

Questionnaires were administered in school classrooms by form tutors who complied with written instructions. The time frame for filling out the questionnaires was 1–1½ school period. Participants could freely choose to participate, and anonymity and confidentially was ensured. As finishing questioning, students sealed themselves the provided envelopes with questionnaires inside. Form tutors reported about the number of participants and process of questioning. The response rate was 84 %.

Upon the completion of the fieldwork, the data were prepared using standard documentation and submitted to the HBSC International Data Bank at the Bergen University, Norway. The data were checked, cleaned, included into the international HBSC database, as well as returned to the country for further statistical processing (*N* = 5730).

The present study includes 3572 students aged 13- and 15-years and who reported about suicide attempts (the proportion of non-reported questions about suicide ranged 2.6–2.8 %). The youngest group of adolescents (11-year-olds) was excluded from the analysis because they were not asked to answer questions about suicide.

### Instrument and measures

We used a standard HBSC international questionnaire adopted after its translation from the Standard English version [42] into Lithuanian and retranslated back into English for approving by the international experts. The questionnaire consists of an mandatory (obligatory) section, that each country is required to include for the production of an international HBSC database, and optional packages, e.g. an optional package “*Family Culture*” [33].

In the present study, the outcome variables were 5 items from the optional package “*Suicide and self-harm*” [43], which source is the Youth Risk Behaviour Survey (YRBS) conducted in the United States [44]. The package of questions introduced the topic of suicide using a short preamble that defines what suicide is, and infers that this is a recognised health problem among people. Students were asked to think about the recent period of 12 months of their life. Questions were then asked in a logical sequence that outlines a causal chain: (1) presence or absence of emotions that stopped doing activities; (2) serious consideration for attempting suicide; (3) making a suicide plan; (4) actual act of attempted suicide; and (5) need of treatment by doctor or nurse. Each question was structured with dichotomous (yes/no) response categories. In the present article the first three outcomes were considered as suicidal ideation (thoughts), and the remaining two were considered as suicidal attempts.

The list of independent variables included gender and age group (13-year-olds and 15-year-olds) of the respondent, as well as a series of the familial variables shortly described in Table 1. More detailed information about these familial variables is provided in our recent publication [38].

**Table 1:** Familial variables

Variable	Origin or source of the variable	*Cronbach*’s Alpha if appropriate	Categories in analysis
Family affluence scale	A set of 6 questions on the material conditions of the households in which children live. The questions cover car ownership, bedroom and bathroom occupancy, holidays, home computers and dishwashing machine [68].		Low (Ref.) Medium High
Family structure	In the list of adult people, the respondents were asked to indicate the persons living in their family.		Intact family (Ref.) Not intact family
Communication with parents^a^	The respondents were asked how easy it is for them to talk to the their father and mother (separately) about things that really bother them [69].		Easy (Ref.) Difficult
Quality of communica-tion within the family	A shortened version of the clear communication scale (4 items) from *Family Dynamics Measure II* [70, 71].	0.87	Good (Ref.) Poor ^#^
Satisfaction with family relationships	The variable was measured by means of an item based on *Cantril*’s [72] ladder, ranged from 0 to 10.		High (7–10 scores) (Ref.) Low (0–6 scores)
Parental monitoring^a^	The measure was based on the scale developed by *Brown* et al. [73], which asks young respondents about how much their father and mother know about five issues of their life.	0.90 (for the father) 0.79 (for the mother)	High (Ref.) Low^#^
Emotional support^a^	The measure was 4-items subscale of the instrument build by *Parker* et al. [74], which is used to assess the quality of parental bonding.	0.84 (for the father) 0.78 (for the mother)	High (Ref.) Low^#^
School-related parental support	The respondents were asked to show how they agree or disagree with the 5 statements on perception of parental emotional support and controlling in various aspects of school [75]. :	0.85	High (Ref.) Low^#^
Parenting style^a^	The measure refers to the strategy developed by *Maccoby* and *Martin* used to assess the four well-known parental disciplinary styles [58].		Authoritative-reciprocal (Ref.) Permissive-indulgent Authoritarian-repressive Permissive-neglectful
Family time together	The evaluation of joint family activity was based on 8 items: (1) watching TV or a video, (2) playing indoor games, (3) eating meals, (4) going for a walk, (5) going places, (6) visiting friends or relatives, (7) playing sports, (8) sitting and talking about things [76].	0.85	Often (Ref.) Rare^#^
Electronic media communication with parents	The respondents were asked to answer how often, in average, they communicate with parents in these ways: (1) speaking by phone; (2) sending SMS messages; (3) writing e-letters; and (4) conversing in real time (e.g. by *Skype*).	0.61	Often (Ref.) Rare^#^
Seeing the parents^a^	The respondents were asked how often they are able to see (meet) their parents because of their job.		See father/mother every day (Ref.) See father/mother not every day

*Ref.* reference category. ^a^ Variables are defined separately for the father and mother. ^#^ Categories correspond to positive and negative factor score in 1-factor analysis of the scale (see Statistical analysis)

### Statistical analysis

Data were analysed in two steps. The first step of analysis was performed within the total sample of 13- and 15-year-olds (*N* = 3572, in order to assess the relationship between suicidal behaviour and family structure only. The second step of analysis was performed within the subsample of those living in intact families (*N* = 2542), and was aimed to explore relationships between suicidal behaviour and a set of variables specific for the intact family.

Reliability analysis with Cronbach’s Alpha measure was used to establish the level of internal consistency of various multi-item scales. Explanatory 1-factor analysis with a principal component analysis was adopted for each scale to construct reliable one-dimensional variables. The factor scores were calculated within subsample of intact families in such way that higher factor scores indicated a higher/better level of family life expected by the respondents. Next, using 0 as a cut-point, factor score values were dichotomized into positive and negative groups, which corresponded to respondents’ inclination for higher and lower scores in the scale.

Associations between familial measures and suicidal ideation forms and suicide attempt were estimated using odds ratios (OR) with 95 % confidence intervals (95 % CI) in a binary logistic regression (BLR) analysis. We used Enter method in multivariable analyses with all variables irrespective of their significance found in a univariable analysis. Interactions between familial variables were tested. Multicollinearity between independent variables in multivariate models was also tested: Tolerance ranged 0.64-0.95, and Variance Inflation Factor (VIF) ranged 1.05-1.55. These estimations did not indicate multicollinearity, therefore pairwise correlations between variables defined for the father and mother showed moderate correlation (e.g., correlation coefficient between father’s monitoring and mothers monitoring was 0.46). All analyses were performed with SPSS (version 20.0; SPSS Inc, Chicago, IL, 2010). *P* < 0.05 was considered statistically significant.

## Results

Table 2 shows demographic and parental characteristics of all studied adolescents and subsample of adolescents living in intact families. The studied sample was sufficiently balanced by gender and age groups both in the total sample and subsample. The distribution of adolescents by their family FAS was skewed towards ‘low’ category. About two-thirds (62.8 %) of the total sample of respondents were regarded as living in intact families. For the items repeated for the father and the mother, there was a significant difference in respondents’ opinion about father’s and mother’s role in their life. Easy communication with the mother was reported more often than with the father (75.9 vs 62.9 %; *P* < 0.001). High level of maternal monitoring was more prevalent than paternal monitoring (62.1 vs 49.3 %; *P* < 0.001). According to the adolescents’ opinion, they can get high emotional support from their mothers more often than from fathers (61.7 vs 57.0 %; *P* = 0.002). Authoritative-reciprocal parenting style the mothers showed more often than the fathers (45.9 vs 41.8 %; *P* < 0.001). Everyday seeing of the mothers was more common than seeing of the fathers (94.0 vs 77.4 %; *P* < 0.001).

**Table 2:** Demographic and parental characteristics of the studied samples

Predictors	No^a^	Percent	*P* ^b^
Total sample, *N* = 3572			
Gender:			
Boys	1805	50.5	
Girls	1767	49.5	
Age:			
13-year-old	1903	53.3	
15-year-old	1669	46.7	
Family FAS:			
Low	1283	36.7	
Medium	1533	43.9	
High	679	19.4	
Family structure:			
Intact family	2454	68.9	
Not-intact family	1118	31.1	
Subsample of respondents living in intact families, *N* = 2454			
Gender:			
Boys	1240	50.5	
Girls	1214	49.5	
Age:			
13 years old	1332	54.3	
15 years old	1122	45.7	
Family FAS:			
Low	766	31.9	
Medium	1084	45.1	
High	553	23.0	
Satisfaction with family relationships:			
High	2095	86.3	
Low	332	13.7	
Communication with the father:			<0.001
Easy	1439	62.9	
Difficult	848	37.1	
Communication with the mother:			
Easy	1741	75.9	
Difficult	553	24.1	
Quality of communication in the family:			
Good	1540	62.8	
Poor	914	37.2	
Father’s monitoring:			<0.001
High	1211	49.3	
Low	1243	50.7	
Mother’s monitoring:			
High	1523	62.1	
Low	931	37.9	
School-related parental support:			
High	1276	52.0	
Low	1178	48.0	
Father’s emotional support:			0.002
High	1400	57.0	
Low	1054	43.0	
Mother’s emotional support:			
High	1514	61.7	
Low	940	38.3	
Father’s parenting style:			<0.001
Authoritative- reciprocal	1008	41.8	
Permissive-indulgent	1001	41.6	
Authoritarian- repressive	188	7.8	
Permissive-neglectful	213	8.8	
Mather’s parenting style:			
Authoritative- reciprocal	1112	45.9	
Permissive-indulgent	1063	43.9	
Authoritarian- repressive	153	6.2	
Permissive-neglectful	96	4.0	
Family time together:			
Often	1148	46.8	
Rare	1306	53.2	
Electronic media communication with parents			
Often	1065	43.4	
Rare	1389	56.6	
Seeing the father			<0.001
Every day	1867	77.4	
Not every day	545	22.6	
Seeing the mother			
Every day	2276	94.0	
Not every day	145	6.0	

^a^ Frequency of missing data is not presented. ^b^ Significance of the difference in respondents’ opinion about the father and the mother (Chi-squared test)

**Fig. 1:**
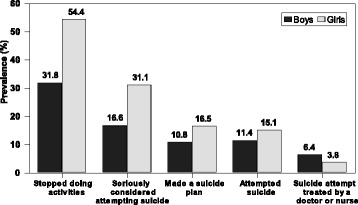
Prevalence of causal chain of suicidal behaviour among adolescents by gender: Lithuanian HBSC survey, 2014

In the total sample of surveyed adolescents, 43.0 % of respondents reported presence of emotions that stopped doing activities during the last 12 months, 23.8 % of respondents seriously considered attempting suicide, 13.7 % of respondents made a suicide plan, and 13.2 % of respondents admitted that they attempted suicide. Some of suicide attempts were serious that needed treatment (4.1 % in total sample or 31.2 % among these who attempted suicide) (Table 3). All forms of suicidal ideation and suicide attempts were more prevalent among girls, while suicide attempts treated by a doctor or nurse were more prevalent among boys (Fig. 1). There was no significant difference in prevalence of suicidal outcomes by age, except attempted suicide, which was more prevalent in the younger 13-year-old age group. The prevalence of reported suicidal ideation seemed significantly higher among adolescents from families of low FAS, and the prevalence of all forms of suicidality was significantly higher among adolescents living in not-intact families (Table 3).

**Table 3:** Prevalence of suicidal ideation and attempts during the last 12 months among 13- and 15-year-old adolescents by gender, age, and family affluence and structure (total sample, *N* = 3572)

	Stopped doing activities			Seriously considered attempting suicide			Made a suicide plan			Attempted suicide			Suicide attempt treated by a doctor or nurse		
	No	%	P*	No	%	P*	No	%	P*	No	%	P*	No	%	P*
Total	1525	43.0		844	23.8		486	13.7		471	13.2		147	4.1	
Gender:															
Boys	568	31,8	**<0.001**	297	16.6	**<0.001**	195	10.9	**<0.001**	205	11.4	**0.001**	91	5.0	**0.005**
Girls	957	54.4		547	31.1		291	16.5		266	15.1		56	3.2	
Age:															
13-year-old	805	42.7	0.690	459	24.4	0.414	248	13.1	0.287	272	14.3	**0.037**	71	3.7	0.217
15-year-old	720	43.3		385	23.2		238	14.3		199	11.9		76	4.6	
Family FAS:															
Low	598	46.8	**0.001**	346	27.1	**0.001**	212	16.6	**<0.001**	187	14.6	0.117	59	4.6	0.538
Medium	644	42.3		341	22.4		179	11.7		191	12.5		60	3.9	
High	255	37.9		135	20.0		78	11.5		79	11.6		25	3.7	
Family structure:															
Intact family	993	40.8	**<0.001**	534	21.9	**<0.001**	299	12.3	**0.001**	277	11.3	**<0.001**	87	3.5	**0.014**
Not-intact family	528	47.8		306	27.8		183	16.6		191	17.2		59	5.3	

* *Z* or Chi-squared test. Significant relations are provided in bold

The univariable and multivariable BLR analyses using data of the total sample and controlling for gender, age and family FAS suggested that broken family was a significant predictor of suicidal ideation and attempts among adolescents (Table 4). Adolescents living in a not-intact family, in comparison with adolescents from an intact family, were significantly more likely to report suicidal behaviour. In a multivariable BLR analysis, for example, the odds for suicidal ideation were increased by 32–35 % (*P* < 0.05), and the odds for attempted suicide were increased by 70 % (*OR* = 1.70, 95 % *CI* 1.38-2.09, *P* < 0.001). The next step of analysis was focussed on the data of intact families. An univariable BLR analysis (Table 5) showed that suicidal ideation and attempted suicide of adolescents living in an intact family were significantly associated with almost all familial predictors included in the present analysis. Among these predictors, satisfaction with family relationships and parental parenting style could be identified as the most important associates. Electronic media communication with parents and seeing the father had any significant association with suicidal outcomes. Suicide attempt treated by a doctor or nurse was found significantly associated with the following familial predictors only: communication with father and mother, quality of communication within the family and family time together. These associations, however, were inverse indicating a lower chance of suicide attempts treated by doctor or nurse among adolescents who reported negative deviances in the mentioned items.

**Table 4:** Association of suicidal ideation and suicide attempts with familial predictors among 13- and 15-year-old adolescents in the total sample of respondents (*N* = 3572): Results from binary logistic regression analysis

Predictors	Stopped doing activities		Seriously considered attempting suicide		Made a suicide plan		Attempted suicide		Suicide attempt treated by a doctor or nurse^b^	
	OR	CI	OR	CI	OR	CI	OR	CI	OR	CI
Univariable analysis										
Gender:										
Boys (Ref.)	1.00		1.00		1.00		1.00		1.00	
Girls	**2.56**	**2.23-2.93**	**2.26**	**1.93-2.66**	**1.62**	**1.34-1.97**	**1.38**	**1.14-1.68**	**0.33**	**0.22-0.50**
Age:										
13-year-old (Ref.)	1.00		1.00		1.00		1.00		1.00	
15-year-old	1.03	0.90-1.17	0.94	0.80-1.10	1.11	0.92-1.34	**0.81**	**0.67-0.99**	**1.75**	**1.18-2.59**
Family FAS:										
Low (Ref.)	1.00		1.00		1.00		1.00		1.00	
Medium	**0.83**	**0.72-0.97**	**0.78**	**0.65-0.92**	**0.67**	**0.54-0.83**	0.83	0.67-1.04	0.99	0.64-1.53
High	**0.69**	**0.57-0.84**	**0.67**	**0.54-0.84**	**0.66**	**0.50-0.87**	0.77	0.58-1.02	1.01	0.57-1.77
Family structure:										
Intact family (Ref.)	1.00		1.00		1.00		1.00		1.00	
Not intact family	**1.33**	**1.15-1.54**	**1.37**	**1.16-1.61**	**1.42**	**1.16-1.74**	**1.63**	**1.34-2.00**	0.98	0.66-1.45
Multivariable analysis^a^										
Gender:										
Boys (Ref.)	1.00		1.00		1.00		1.00		1.00	
Girls	**2.61**	**2.27-3.00**	**2.35**	**1.99-2.77**	**1.75**	**1.43-2.14**	**1.42**	**1.16-1.74**	**0.31**	**0.21-0.48**
Age:										
13-year-old (Ref.)	1.00		1.00		1.00		1.00		1.00	
15-year-old	1.04	0.91-1.20	0.93	0.79-1.09	1.08	0.89-1.32	**0.80**	**0.65-0.97**	**1.85**	**1.22-2.82**
Family FAS:										
Low (Ref.)	1.00		1.00		1.00		1.00		1.00	
Medium	0.89	0.76-1.04	0.83	0.69-0.99	**0.72**	**0.58-0.89**	0.89	0.72-1.11	0.97	0.61-1.53
High	**0.77**	**0.63-0.94**	**0.74**	**0.59-0.94**	**0.74**	**0.55-0.98**	0.88	0.66-1.17	1.05	0.57-1.93
Family structure:										
Intact family (Ref.)	1.00		1.00		1.00		1.00		1.00	
Not intact family	**1.32**	**1.13-1.53**	**1.35**	**1.13-1.60**	**1.34**	**1.09-1.65**	**1.70**	**1.38-2.09**	1.03	0.67-1.59

^a^ Method = Enter. ^b^ Results from analysis of subsample attempted suicide. *Ref.* reference category, *OR* Odds Ratio, *CI* 95 % Confidence Interval. Significant relations are provided in bold

**Table 5:** Association of suicidal ideation and suicide attempts with familial predictors among 13- and 15-year-old adolescents in the subsample of respondents living in intact families (*N* = 2542): Results from univariable binary logistic regression analysis

Predictors	Stopped doing activities		Seriously considered attempting suicide		Made a suicide plan		Attempted suicide		Suicide attempt treated by a doctor or nurse^a^	
	OR	CI	OR	CI	OR	OR	OR	CI	OR	CI
Gender:										
Boys (Ref.)	1.00		1.00		1.00		1.00		1.00	
Girls	**2.64**	**2.23-3.11**	**2.16**	**1.77-2.63**	**1.46**	**1.15-1.87**	1.18	0.92-1.51	**0.33**	**0.19-0.56**
Age:										
13-year- old (Ref.)	1.00		1.00		1.00		1.00		1.00	
15-years-old	0.99	0.95-1.17	0.93	0.76-1.12	1.25	0.98-1.59	0.80	0.62-1.03	**2.06**	**1.23-3.45**
Family FAS:										
Low (Ref.)	1.00		1.00		1.00		1.00		1.00	
Medium	**0.82**	**0.68-0.98**	**0.77**	**0.62-0.95**	**0.60**	**0.45-0.79**	0.86	0.64-1.15	0.80	0.44-1.43
High	**0.70**	**0.56-0.87**	**0.64**	**0.49-0.84**	**0.64**	**0.46-0.89**	0.86	0.61-1.22	0.84	0.42-1.70
Father’s monitoring:										
High (Ref.)	1.00		1.00		1.00		1.00		1.00	
Low	**1.64**	**1.39-1.93**	**1.63**	**1.34-1.98**	**1.83**	**1.43-2.35**	**1.50**	**1.17-1.94**	0.72	0.43-1.20
Mother’s monitoring:										
High (Ref.)	1.00		1.00		1.00		1.00		1.00	
Low	**1.40**	**1.19-1.65**	**1.57**	**1.29--1.90**	**1.92**	**1.50-2.45**	**1.85**	**1.44-2.38**	0.96	0.58-1.60
Satisfaction with family relationships:										
High (Ref.)	1.00		1.00		1.00		1.00		1.00	
Low	**3.70**	**2.89-4.74**	**3.54**	**2.77-4.53**	**3.49**	**2.63-4.63**	**2.84**	**2.11-3.83**	0.83	0.46-1.50
Communication with father:										
Easy (Ref.)	1.00		1.00		1.00		1.00		1.00	
Difficult	**1.99**	**1.68-2.37**	**1.98**	**1.62-2.42**	**1.72**	**1.33-2.22**	**1.53**	**1.17-1.99**	**0.34**	**0.19-0.61**
Communication with mother:										
Easy (Ref.)	1.00		1.00		1.00		1.00		1.00	
Difficult	**1.75**	**1.45-2.13**	**1.79**	**1.44-2.23**	**2.15**	**1.65-2.81**	**1.77**	**1.34-2.34**	**0.51**	**0.28-0.93**
Quality of communication within the family:										
Good (Ref.)	1.00		1.00		1.00		1.00		1.00	
Poor	**1.87**	**1.59-2.22**	**1.97**	**1.62-2.40**	**2.17**	**1.70-2.77**	**2.01**	**1.56-2.59**	**0.56**	**0.34-0.94**
School-related parental support:										
High (Ref.)	1.00		1.00		1.00		1.00		1.00	
Low	**1.44**	**1.22-1.69**	**1.66**	**1.37-2.01**	**1.66**	**1.30-2.12**	**1.91**	**1.48-2.47**	1.07	0.64-1.81
Father’s emotional support:										
High (Ref.)	1.00		1.00		1.00		1.00		1.00	
Low	**1.65**	**1.40-1.94**	**1.94**	**1.60-2.36**	**2.16**	**1.69-2.76**	**1.78**	**1.38-2.29**	0.70	0.42-1.16
Mother’s emotional support:										
High	1.00		1.00		1.00		1.00		1.00	
Low	**1.61**	**1.37-1.90**	**1.94**	**1.60-2.36**	**2.26**	**1.77-2.89**	**1.88**	**1.46-2.42**	0.86	0.52-1.43
Father’s parenting style:										
Authoritative- reciprocal (Ref.)	1.00		1.00		1.00		1.00		1.00	
Permissive-indulgent	1.11	0.93-1.34	0.98	0.79-1.23	0.91	0.64-1.28	0.92	0.69-1.24	0.89	0.48-1.63
Authoritarian- repressive	**2.58**	**1.88-3.56**	**2.15**	**1.53-3.02**	**2.84**	**1.92-4.16**	**1.96**	**1.28-3.00**	0.64	0.26-1.57
Permissive-neglectful	**2.01**	**1.49-2.72**	**1.71**	**1.22-2.38**	**1.64**	**1.09-2.48**	**1.87**	**1.24-2.81**	1.13	0.51-2.50
Mother’s parenting style:										
Authoritative- reciprocal (Ref.)	1.00		1.00		1.00		1.00		1.00	
Permissive-indulgent	**1.23**	**1.04-1.46**	1.05	0.85-1.29	1.08	0.82-1.42	0.93	0.70-1.23	0.87	0.49-1.55
Authoritarian- repressive	**2.16**	**1.53-3.04**	**2.53** ^**c**^	**1.77-3.63**	**3.43**	**2.29-5.15**	**1.94**	**1.24-3.06**	0.55	0.21-1.48
Permissive-neglectful	**2.03**	**1.34-3.09**	**2.09**	**1.74-4.16**	**3.48**	**1.74-4.16**	**3.39**	**2.09-5.51**	1.19	0.50-2.85
Family time together:										
Often (Ref.)	1.00		1.00		1.00		1.00		1.00	
Rare	**1.64**	**1.39-1.93**	**1.38**	**1.14-1.68**	1.22	0.95-1.55	0.99	0.77-1.28	**0.47**	**0.28-0.79**
Electronic media communication with parents										
Often (Ref.)	1.00		1.00		1.00		1.00		1.00	
Rare	0.89	0.77-1.05	0.99	0.82-1.20	0.90	0.70-1.14	0.87	0.67-1.11	0.85	0.51-1.41
Seeing the father										
Every day (Ref.)	1.00		1.00		1.00		1.00		1.00	
Not every day	1.17	0.96-1.42	0.96	0.79-1.25	0.97	0.73-1.31	0.93	0.68-1.26	0.71	0.37-1.36
Seeing the mother										
Every day (Ref.)	1.00		1.00		1.00		1.00			
Not every day	1.31	0.93-1.83	**1.47**	**1.01-2.13**	1.32	0.83-2.12	**1.71**	**1.09-2.69**	1.02	0.42-2.46

^a^ Results from analysis of subsample attempted suicide. *Ref.* reference category, *OR* Odds Ratio, *CI* 95 % Confidence Interval. Significant relations are provided in bold

In a multivariable BLR analysis (Table 6), adjusting data for gender, age and family FAS, the increased odds for suicidal behaviour were revealed among adolescents who reported low satisfaction with family relationships, low father’s and mother’s emotional support (significant for seriously considered attempting suicide and made a suicide plan only), low mother’s monitoring and low school-related parental support (significant for attempted suicide only), and authoritarian-repressive or permissive-neglectful father’s parenting style and permissive-neglectful mother’s parenting style (significant for most of the suicidal outcomes). Rare family time together (significant for attempted suicide only) and rare electronic media communication with parents (significant for stopped doing activities and attempted suicide) seemed to be protective determinants against suicidal behaviours. Suicide attempts treated by doctor or nurse were significantly associated with gender (girls were less likely than boys), and age (13-year-olds were less likely than 15-year-olds), as well as with family time together (adolescents who reported often activities together with their family were more likely than their counterparts).

**Table 6:** Association of suicidal ideation and suicide attempts with familial predictors among 13- and 15-year-old adolescents in the subsample of respondents living in intact families (*N* = 2542): Results from multivariable binary logistic regression analysis^a^

Predictors	Stopped doing activities		Seriously considered attempting suicide		Made a suicide plan		Attempted suicide		Suicide attempt treated by a doctor or nurse^b^	
	OR	CI	OR	CI	OR	OR	OR	CI	OR	CI
Gender:										
Boys (Ref.)	1.00		1.00		1.00		1.00		1.00	
Girls	**2.60**	**2.12-3.19**	**2.33**	**1.81-2.99**	**1.69**	**1.23-2.31**	**1.38**	**1.01-1.91**	**0.21**	**0.09-0.47**
Age:										
13-year- old (Ref.)	1.00		1.00		1.00		1.00		1.00	
15-years-old	0.89	0.73-1.08	**0.79**	**0.63-0.99**	1.17	0.87-1.56	**0.72**	**0.53-0.98**	**3.20**	**1.52-6.72**
Family FAS:										
Low (Ref.)	1.00		1.00		1.00		1.00		1.00	
Medium	1.05	0.84-1.30	1.01	0.78-1.30	0.75	0.54-1.04	1.09	0.76-1.54	0.56	0.24-1.29
High	0.92	0.71-1.20	0.86	0.62-1.18	0.89	0.60-1.31	1.12	0.75-1.68	0.83	0.32-2.16
Father’s monitoring:										
High (Ref.)	1.00		1.00		1.00		1.00		1.00	
Low	1.19	0.94-1.49	1.15	0.87-1.52	1.32	0.92-1.88	0.98	0.68-1.42	1.44	0.58-3.59
Mother’s monitoring:										
High (Ref.)	1.00		1.00		1.00		1.00		1.00	
Low	1.20	0.95-1.52	1.20	0.92--1.57	1.16	0.83-1.63	**1.42**	**1.01-2.02**	0.94	0.40-2.22
Satisfaction with family relationships:										
High (Ref.)	1.00		1.00		1.00		1.00		1.00	
Low	**2.57**	**1.87-3.53**	**2.03**	**1.47-2.79**	**1.57**	**1.06-2.31**	**2.00**	**1.37-3.00**	1.29	0.52-3.16
Communication with father:										
Easy (Ref.)	1.00		1.00		1.00		1.00		1.00	
Difficult	1.09	0.86-1.37	1.10	0.84-1.44	0.96	0.68-1.36	1.14	0.79-1.69	0.64	0.26-1.56
Communication with mother:										
Easy (Ref.)	1.00		1.00		1.00		1.00		1.00	
Difficult	1.26	0.98-1.63	1.15	0.86-1.55	1.39	0.97-1.99	1.16	0.80-1.69	0.70	0.27-1.78
Quality of communication within the family:										
Good (Ref.)	1.00		1.00		1.00		1.00		1.00	
Poor	1.01	0.81-1.27	1.05	0.80-1.37	1.26	0.89-1.78	1.26	0.88-1.81	0.58	0.23-1.47
School-related parental support:										
High (Ref.)	1.00		1.00		1.00		1.00		1.00	
Low	1.05	0.85-1.31	1.15	0.88-1.50	0.82	0.58-1.16	**1.42**	**1.00-2.03**	1.41	0.56-3.52
Father’s emotional support:										
High (Ref.)	1.00		1.00		1.00		1.00		1.00	
Low	1.04	0.82-1.31	**1.35**	**1.03-1.78**	**1.55**	**1.08-2.21**	1.19	0.82-1.72	0.93	0.37-2.34
Mother’s emotional support:										
High	1.00		1.00		1.00		1.00		1.00	
Low	1.10	0.87-1.39	**1.32**	**1.01-1.74**	**1.55**	**1.09-2.21**	1.33	0.93-1.92	1.39	0.58-3.34
Father’s parenting style:										
Authoritative- reciprocal (Ref.)	1.00		1.00		1.00		1.00		1.00	
Permissive-indulgent	1.05	0.84-1.32	1.02	0.78-1.34	0.90	0.64-1.28	0.97	0.68-1.40	0.51	0.21-1.26
Authoritarian- repressive	**1.91**	**1.30-2.82**	1.38	0.91-2.08	**1.64**	**1.01-2.65**	1.55	0.92-2.58	0.74	0.21-2.54
Permissive-neglectful	**1.53**	**1.03-2.26**	1.05	0.68-1.62	0.67	0.38-1.18	1.09	0.62-1.90	1.04	0.25-4.17
Mother’s parenting style:										
Authoritative- reciprocal (Ref.)	1.00		1.00		1.00		1.00		1.00	
Permissive-indulgent	0.99	0.79-1.23	0.86	0.66-1.13	0.97	0.69-1.37	0.85	0.60-1.20	1.60	0.65-3.90
Authoritarian- repressive	1.08	0.69-1.67	1.22	0.77-1.93	1.57	0.93-2.66	0.86	0.48-1.55	1.07	0.26-4.46
Permissive-neglectful	1.11	0.62-1.97	**1.89**	**1.04-3.46**	**3.48**	**1.76-6.87**	**2.36**	**1.19-4.60**	0.51	0.11-2.47
Family time together:										
Often (Ref.)	1.00		1.00		1.00		1.00		1.00	
Rare	1.19	0.96-1.47	0.88	0.67-1.14	0.72	0.51-1.01	**0.61**	**0.43-0.87**	**0.39**	**0.17-0.90**
Electronic media communication with parents										
Often (Ref.)	1.00		1.00		1.00		1.00		1.00	
Rare	**0.79**	**0.65-0.96**	0.89	0.71-1.12	0.79	0.59-1.08	**0.73**	**0.54-0.99**	1.12	0.54-2.32
Seeing the father										
Every day (Ref.)	1.00		1.00		1.00		1.00		1.00	
Not every day	1.17	0.93-1.47	1.03	0.79-1.35	0.97	0.69-1.37	0.90	0.63-1.29	0.47	0.17-1.32
Seeing the mother										
Every day (Ref.)	1.00		1.00		1.00		1.00			
Not every day	1.36	0.91-2.03	1.42	0.91-2.22	1.14	0.65-2.00	1.52	0.89-2.61	0.54	0.13-2.31

^a^ Method = Enter. ^b^ Results from analysis of subsample attempted suicide. *Ref.* reference category, *OR* Odds Ratio, *CI* 95 % Confidence Interval. Significant relations are provided in bold

## Discussion

This paper draws on recent Lithuanian data from the World Health Organization cross-national HBSC study, which investigates a range of familial determinants on youth health and heath behaviour [33]. We aimed to analyze the predictive value of familial variables on suicidal behaviour among adolescents in Lithuania. Suicidal behaviour was the main focus due to still high suicide rate both for the whole population and for young people in the country [5, 7, 8]. In addition, the rationale for this study arose from the significant family transformations over last two decades that were the consequence of a swift transition away from a totalitarian regime to a democratic society in Central and Eastern European countries, including Lithuania [29].

The associations between parental and familial factors and adolescent maladjusted behaviour have been extensively examined [34]. Recent systematic reviews described the role of family functioning and parenting on adolescents suicidal behaviour and confirmed the important protective role of positive family processes [22, 24, 45]. In our study, besides family structure, the statistical significance of associations was assessed for at least 15 variables, which measured different aspects of child–parent relationships. In order to avoid overestimation of the specific father’s or mother’s role in single-parent and step-parent families, an intact family was selected as a model to obtain valid findings. Partly, this decision was supported by *Recker* research [46].

The present study revealed that only 62.8 % of the total sample of respondents were living in intact families, whereas two decades ago, in 1994, during the first HBSC study wave in Lithuania, the corresponding figure was significantly greater - 82.7 % [47]. A broken family was shown previously linked to the risk for engaging adolescents in suicide attempts or other risk-taking behaviour [21, 26, 28, 48, 49]. The results of Garnefski and Diekstra [50] study, published two decades ago, have already indicated that adolescents from single-parent and step-parent families reported lowered self-confidence, hightened anxiety and loneliness, more depressed mood, more suicidal thoughts, and even more attempts to commit suicide than children from intact families. Our study was in accordance with the literature indicating that adolescents who did not live with both parents were at least 30 % more likely to express suicidal thoughts and at least 70 % more likely to report attempted suicide than their counterparts. This finding suggests suicide attempts in adolescents can be associated with negative experiences of parental divorce and loss of social support in single-parent family, which can be seen as critical life events. Other studies that addressed adolescent suicide attempts support this assumption [21, 49].

It is well known that good parent–child relations and communication is a key determinant for good psychological well-being and positive behaviour among young people [36, 38, 40]. The studies claim that good communication with mother and with father is associated with less risk for suicidal behaviour [28, 34]. Our previous studies which were based on the data of HBSC surveys in 2002 [51] and 2010 [35] confirmed these associations as a consistent pattern for both boys and girls and with respect to both parents. In these studies we found that easy communication with parents is a more robust barrier to suicidal behaviour than living with both parents. In line with other studies [37], the present study showed that adolescents find it easer to talk to their mother rather than to their father. In univariable BLR analysis we found that difficult communication with father and mother significantly increased the likelihood for all manifestations of suicidal behaviour (here and below: except suicide attempt treated by a doctor or nurse). But these two variables were no longer significant predictors in multivariable BLR analysis.

In the univariable analysis, we observed a significant relationship between suicidal behaviour and most of other studied familial variables. Some of them retained their significance running the multivariable analysis: low father’s and mother’s emotional support increased the odds for seriously consideration to attempt suicide and making a suicide plan, low mother’'s monitoring and school-related parental support increased the odds for attempted suicide. Adolescent’s satisfaction with family relationships was the strongest predictor for suicidal behaviour: having low satisfaction with family relationships increases the odds for suicide attempts by 2 times. Our recent study [38] has also demonstrated a high predictive value of this variable in regard to current smoking of adolescents. These findings suggest that the measure of subjective satisfaction with family relationships integrates adolescent’s feelings about all positive family processes, including parental social and psychological support. This a relatively new result is in accordance with other studies, which have shown that parental support and affection serve as factor that protect against suicide attempts [34, 52, 53].

In this study parenting style was in focus too. Research has firmly shown that adolescents reared within authoritative families have better scores in several areas, while adolescents reared in authoritarian and negligent families have higher developmental risks and problems, increased impulsiveness, delinquency and be more willing to engage in early risk behaviours such as substance use [33, 54]. However, concerning suicidal behaviour and parenting style, the literature is scarse. The negative effect of authoritarian [55–57] and rejecting-neglecting [21] parenting has been alredy discussed, but we still accessed a predictive value of parenting styles. In contrast with other authors, we used four-styles categorization of parenting, developed by Maccoby and Martin [58]. In addition, to avoid a big overlap between styles and to clarify disciplinary styles we redused the scale proposed for the HBSC survey [33] to one question, which was repeated for the father and mother. Our analysis conducted with multivariable BLR analysis revealed the increased odds for suicidal behaviour among adolescents with their father’s authoritarian-repressive parenting style (significant association with stopped doing activities and made a suicide plan). In respect of mother’s parenting, an increase in odds for suicidal behaviour was significant when permissive-neglectful parenting style occured. This relatively new finding distinguished the effect of paternal and maternal roles in disciplinary parenting of adolescents.

In present study we also hypothesized that frequent activities together with parents, frequent interactions with parents by phone or using other electronic media, and, finally, seeing the parents at least daily can facilitate positive communication with parents, as well as can play a helpful role in adolescent’s satisfaction with family relationships. Consequently, frequent communication with the parents should play a protective role [33, 59, 60]. However, the results of multivariable BLR analysis in our study indicated that often family time together and often communication with parents using electronic media significantly increased the odds for attempted suicide (by 64 and 37 % correspondingly). It is not easy to explain these relatively new result concerning above described processes in family as no other studies investigating such associations were found.

Finally, suicide attempts that resulted in an injury, poisoning or overdose that had to be treated by a doctor or nurse is the last link in a causal chain to suicide. It is well established that prevalence of suicide and suicide-related behaviour increases with age and a gender paradox exists with regard to youth suicidal behaviour: i.e. while suicide rates are higher among boys than girls, girls are more likely have suicidal ideation and to attempt suicide [17]. The results of our study were in line with the above statment, while the YRBS survey in the US (2013) [44] reported the rate of having made a suicide attempt that had to be treated by a doctor or nurse higher among girls (3.6 %) than boys (1.8 %).

According to our data, the prevalence of “serious” suicide attempts was significantly higher among adolescents living in non-intact families comparing with their peers from intact families, but the family structure was no longer a significant predictor for such suicide attempts in multivariable BLR analysis. The predictive value of other familial variables in regard to suicide attempts treated by a doctor or nurse was analyzed among adolescents liwing in intact families, but this analysis was conducted within the subgroup of adolescents who reported attempted suicide. Such approach could be comparable with the clinical studies of patients attempted non-fatal suicide [61, 62], but the literature that presents resuls of clinical studies addressed familial factors is scarse [62, 63]. In this respect, the results of our study can intrigue clinicians.

Due to small number of respondents who reported suicide attempts treated by a doctor or nurse, few associations between this suicidal outcome and familial predictors were significant.. In contrast with the above presented results addressed suicidal ideation and attempted suicide, most of revealed associations had an inverse tendency: adolescents from families with better functioning were more likely to attempt suicide treated by a doctor or nurse than adolescents from families with poorer functioning. For example, adolescents who reported often family time together were at 2.5 time higher odds of “serious” suicide attempt than they peers, who reported less often family time together. It is difficult to explain these findings, therefore, there is a need for further research to confirm them as no other studies investigating such associations were found.

The current study, in line with the findings of other studies in this field [21, 45, 56], suggests that it is essential to consider family life practices in planning intervention programs for prevention of suicides among adolescents. This challenge requires new understanding and innovative approaches towards youth mental health care and promotion. In 2007 the Parliament of Republic of Lithuania approved the “National Mental Health Strategy”, which is being achieved through innovative mental health promotion/prevention state programmes [64]. However, only a small number of services are provided at community level, including activities in families. Most of preventive mental health programmes for the young population are implemented by non-govermental organizations, and due to absence of the system of state funding to underpin preventive mental health services for adolescents prevention initiatives in Lithuania are fragmented [65, 66].

### Study strengths and limitations

This study has a number of strengths. A large, nationally representative non-clinical sample and high participation rate in the survey could be considered as the strengths of primary importance of the current study. It is also important that our research was a part of the cross-national collaborative HBSC study. The application of standardized methods including the HBSC questionnaire, which was developed by international experts, is another advantage of this study. The measures of family life were based on valid scales. The internal reliabilities (*Cronbach*’s alphas) for the applied scales were quite high. The study controls for family affluence in examining the effect of familial variables. Given the extensive debate over the role of the family life on offspring suicidal behaviour, the results of our study are also a step forward to filling the gap in existing research.

Study limitations require consideration too. As with all cross-sectional studies, the HBSC is based on respondent self-report that can be affected by recall bias and social desirability. Self-reported suicide attempts might not represent the group of actual suicide attempters comprehensively. It is also unclear how the set of established associations manifests among those who committed suicide. However, self-reports are commonly used in large epidemiological studies. The HBSC questionnaire survey as well as other similar studies carried on adolescents health behaviour presents an example of very sensitive and personal issues for investigation such as suicide attempts. To cope with this source of a potential bias of self-reporting, special attempts were made by researchers to provide warranty of anonymity and confidentiality. In addition, the questions were subject to piloting and pre-testing at international and national levels prior to the main survey [33, 41].

In present study the analysis of associations between suicidal and familial variables was limited within intact families, in general. The reasons for such approach were methodological constraints that limited application of selected measures in non-intact families if they were specified for the father and the mother. For instance, the data of the present survey demonstrated that 1046 (28.4 %) respondents were living in a family without the father, therefore, in this group 426 (40.7 %) respondents reported how easy is it to communicate with their fathers, or 633 (60.5 %) respondents indicated how much their fathers know about their activities. Such disparities can be naturally occurred as the family is divorced, but the child is able to meet his father, for example. However, the described cases are confusing in regard of the simple definition of family structure and the further studies are need to explore non-intact families. The study did not include information related to parents’ suicide history, which may play an important role in predicting suicidal behaviour of young people, as the questionnaire did not include related questions. It might be taken into consideration, as well, that the concept family time together itself may cover a wide majority of different meanings. The same length of family time together may have different effects on a child depending on control, limitations, closeness or openness of relationships within family.

The study also did not include information related to school or other places, which may play an important role in determining suicidality of young people, as this is outside limits of the present study. Nevertheless, our study focussed on the specific psychosocial familial determinants and their impact on young people suicide risk, and its results provide directions for suicide prevention efforts intervening at family level.

A possible source of bias could operate throughout unmeasured confounding variables. While analysis of data was adjusted for family affluence, information was not available on all potential confounders, i.e., common causes of both familial predictors and adolescents’ suicidal behaviour, such as parents’ education, family conflicts, psychotic-like experiences and other variables. Assessment of the impact of unmeasured confounders needs special methods, however, this was not investigated in the present study.

Finally, given the cross-sectional design of this study with a rather exploratory nature, we should be careful with interpreting causality. Thus, more studies, including studies with a longitudinal design, are needed to confirm the results and to establish scientific evidence on the relationships found in this study. If these results are confirmed, parents should be advised to apply the more positive approach in parenting and managing their parental roles by helping their children to achieve certain goals.

## Conclusions

The current study suggests that the young people of Lithuania are at particular risk for mental health problems such as suicides. The findings confirmed the hypothesis that a non-intact family structure, weak child–parent relationships and contact, lack of parental control, and other familial variables are significant predictors of suicidal ideation (stopped doing activities, considered suicide, planned suicide) and attempts among adolescents of Lithuania. It is essential to consider family life practices in planning intervention programs for prevention of suicides among adolescents.

## Abbreviations

BLR, binary logistic regression; CI, confidence interval; FAS, family affluence scale; HBSC, Health Behaviour in School-aged Children, a World Health Organization cross-national study; N, number of respondents; OR, odds ratio; YRBS, Youth Risk Behaviour Survey
